# Evaluating Pilocarpine 1% Eye Drops in Presbyopia: A Triple‐Blinded Randomized Trial

**DOI:** 10.1155/joph/8334950

**Published:** 2026-07-27

**Authors:** Mohsen Gohari, Mohammad Hosein Abootorabi, Fateme Heidari

**Affiliations:** ^1^ Ophthalmologist, Shahid Sadoughi University of Medical Sciences, Yazd, Iran, ssu.ac.ir; ^2^ Ophthalmologist, Shahid Sadoughi University of Medical Sciences and Health Services, Yazd, Iran, ssu.ac.ir; ^3^ Department of Medicine, School of Medical Science, Yazd Branch, Islamic Azad University, Yazd, Iran, azad.ac.ir

**Keywords:** pilocarpine, presbyopia, randomized trial, triple blind

## Abstract

**Background:**

Presbyopia is an age‐related condition that affects near vision and daily activities. Although several correction methods are available, each has certain limitations, leading to interest in pharmacologic alternatives such as pilocarpine eye drops. This study evaluates the safety and clinical effectiveness of 1% pilocarpine eye drops, as data on its use remain limited.

**Material and Method:**

A triple‐blinded clinical trial was conducted in 2024 in Yazd, Iran, enrolling 60 presbyopic patients aged 40–60 years. The study was approved by the Iranian Registry of Clinical Trials. Participants received either 1% pilocarpine eye drops or a placebo daily for 30 days. Their visual acuity—both near and distance—was assessed under mesopic conditions at six intervals. Throughout the trial, any side effects and treatment satisfaction were monitored. Statistical analysis was performed, considering results significant if *p* < 0.05.

**Results:**

In this study, the treatment group demonstrated a significant improvement in uncorrected near visual acuity (UNVA) at all follow‐ups compared to the placebo group, peaking 1 hour after treatment (*p* ≤ 0.001). Although a significant change in uncorrected distance visual acuity (UDVA) from baseline was observed within the treatment group, no significant between‐group difference in UDVA was found at the 30‐day follow‐up. Overall, patient satisfaction was significantly higher in those receiving pilocarpine (*p* ≤ 0.001).

**Discussion:**

This study supports previous research by showing improvements in UNVA, a quick onset of effects, and an acceptable safety profile. However, there was a slight worsening in UDVA; this may be due to higher initial measurements or temporary effects. Unlike many past trials, this study utilizes a rigorous triple‐blinded design and measures effects at six different times, giving a thorough look at both the onset and duration of effects.

**Conclusion:**

A 1% pilocarpine treatment has been found to significantly improve near vision in presbyopic patients, with minimal side effects and high patient satisfaction.

**Trial Registration:** Iranian Registry of Clinical Trials (IRCT): IRCT20240325061363N1

## 1. Introduction

Presbyopia is a common age‐related condition that causes difficulty focusing on near objects as the eye gradually loses its ability to accommodate. The loss of accommodation is likely because of ciliary muscles’ weakening and changes in the crystalline lens with increasing age [1, 2]. Presbyopia causes limitation in daily activities such as reading fine print, seeing fine details on nearby objects, or threading a needle and can present with a need for increased lighting, epiphora, or diplopia [3]. Therefore, this condition affects patients’ emotional well‐being and deteriorates their quality of life [4].

A study by Fricke et al. estimates that 1.8 billion people worldwide suffer from presbyopia, underscoring the need for practical, safe, and convenient treatment options [5]. Several corrective options are currently available for presbyopia, including reading glasses, bifocal or multifocal progressive lenses, contact lenses, and various surgical procedures [6, 7]. Although these methods can effectively improve near vision, each is associated with specific limitations. Glasses may restrict the visual field or cause discomfort; contact lenses are often associated with dryness and inconvenience; and surgical options carry a risk of complications [8]. Therefore, pharmacological interventions have recently gained attention as a noninvasive alternative [2, 9, 10].

Pilocarpine is a cholinergic agonist that primarily acts on muscarinic M3 receptors. Its effect on near vision appears to occur through two mechanisms. By inducing contraction of the iris sphincter, pilocarpine causes pupillary constriction, creating a pinhole effect that increases the depth of focus. In addition, stimulation of the ciliary muscle enhances accommodative function. Collectively, these actions support pilocarpine as a noninvasive pharmacological option for the management of presbyopia [8, 9].

Although pilocarpine has been widely studied for the treatment of presbyopia, there are still important aspects we need to understand better. Questions about its long‐term effectiveness, any potential impact on distance vision, and how well patients tolerate using it regularly remain. In this context, a triple‐blinded randomized clinical trial was conducted, with multiple time points. This study aimed to provide a thorough evaluation of how effective and safe 1% pilocarpine eye drops are for people dealing with presbyopia.

## 2. Material and Method

This triple‐blinded randomized clinical trial was conducted in an outpatient ophthalmology department in Yazd, Iran, in 2024. The study was prospectively approved by the Institute Ethics Committee (IR.SSU.MEDICINE.REC.1402.405) and the Iranian Registry of Clinical Trials. The study protocol followed the ethical principles of the Declaration of Helsinki, and informed consents were obtained from all participants.

Participants were consecutively recruited from the outpatient ophthalmology clinic among patients presenting with age‐related near vision impairment affecting daily activities. Individuals aged between 40 and 60 years, in good health, and exhibiting both objective and subjective signs of presbyopia were included. A thorough ocular evaluation was conducted to evaluate exclusion criteria. The evaluation included the followings:

1. Medical history review: A thorough interview was conducted to assess for ocular surgery history, eye injuries, severe dry eye conditions, or allergy to eye drops. Participants were also screened for the use of medications that could affect accommodation or pupil size, including psychotropic medications, antidepressants, anticholinergic agents, antihistamines, and other topical ocular medications with potential pupillary effects. Individuals presenting with any of these factors were excluded from the study.
2. Slit‐lamp examination: A slit‐lamp examination was performed using a Haag–Streit device to assess the anterior segment and exclude conditions such as corneal opacities, cataracts, congenital pupillary anomalies, and other anterior chamber pathologies.
3. Fundoscopy: Fundoscopic examination was performed using an indirect ophthalmoscope (Heine Omega 500) to take a close look at the retina and optic disc. Patients with retinal issues, including macular degeneration, retinal detachment, or trauma‐related retinal changes, were excluded from the study.
4. Intraocular pressure (IOP) measurement: IOP was evaluated using a noncontact tonometer (Goldmann applanation tonometer) to identify and exclude participants who might have glaucoma or abnormal levels of IOP.
5. Visual acuity testing: Uncorrected distance visual acuity (UDVA) and uncorrected near visual acuity (UNVA) were measured using a Snellen chart to identify significant refractive errors or visual impairments, including high myopia or amblyopia.

Before randomization, baseline visual function was assessed, and no significant differences in UNVA or UDVA were found between the study groups. A total of 60 participants were randomly assigned in a 1:1 ratio to either the intervention group (*n* = 30) or the control group (*n* = 30) using a random number table. The randomization process achieved balanced baseline demographic and visual characteristics, as no statistically significant differences were identified between groups with respect to age, sex, baseline UNVA, or baseline UDVA.

The intervention group received 1% pilocarpine eye drops (Sinadarou Co., Iran), while the control group received artificial tears (Sinadarou Co., Iran). To keep things fair and unbiased, both eye drops were packaged identically as A or B by the hospital pharmacist, who was the only person aware of the assignments and did not participate in data collection or analysis. This setup helped ensure triple blinding of participants, investigators, and the statistician. The use of assigned interventions was indicated bilaterally daily, at 9 a.m. for 30 days.

The patients were visited at baseline, 20 min, 1 h, 6 h, 14 days, and 30 days’ time points. At baseline, participants underwent visual acuity assessment before instillation of the study medication. Following the first administration, binocular UNVA and UDVA were assessed at 20 min, 1 h, and 6 h. At the 14‐day and 30‐day follow‐up visits, visual acuity was measured approximately 1 h after instillation. During all visits, the binocular vision UNVA and UDVA were assessed using Snellen’s chart at distances of 33 cm and 6 m, respectively, under mesopic light conditions (approximately 10 lux). Binocular visual acuity values recorded using Snellen charts were converted to the logarithm of the minimum angle of resolution (log MAR) units using a standard conversion table for statistical analysis.

Additionally, participants were examined at each follow‐up visit and asked about any treatment‐related adverse effects, including burning sensation, flashes of light, headache, and other ocular or visual symptoms. Any adverse event reported at least once during the treatment period was recorded and included in the analysis. Patient satisfaction was assessed at the 30‐day follow‐up visit using a four‐category subjective rating scale (poor, moderate, good, and very good).

Statistical analyses were conducted using IBM SPSS Statistics for Windows, Version 25.0 (IBM Corp., Armonk, NY, USA). Quantitative variables are expressed as mean ± standard deviation, while qualitative variables are presented as frequencies and percentages. Independent‐sample *t*‐tests were utilized for between‐group comparisons, while chi‐square or Fisher’s exact tests were used for categorical data. Repeated measures ANOVA with Bonferroni correction assessed within‐group time effects and interactions. Paired *t*‐tests evaluated intragroup differences. Between‐group differences are reported with two‐sided 95% confidence intervals, and a *p* value of less than 0.05 denotes statistical significance for all two‐tailed tests.

## 3. Result

A total of 60 patients diagnosed with presbyopia fulfilled the inclusion and exclusion criteria and enrolled in this triple‐blinded randomized clinical trial. The participants were equally randomized into two intervention and control groups, which received pilocarpine 1% and placebo, respectively. All participants completed the 30‐day follow‐up period. The mean (± SD) age of patients was 50.87 (± 5.37), ranging between 43 and 59 years; among them, 57% were male. As provided in Table 1, demographics were generally balanced between the two groups.

**TABLE 1:** Demographic characteristics of study population.

Variable		Control group	Intervention group	Total	*p*
Age		50.93 (5.27)	50.80 (5.55)	50.87 (5.37)	0.924

Gender	Male	16 (53.0%)	18 (60.0%)	34 (57.0%)	0.602
	Female	14 (47.0%)	12 (40.0%)	26 (43.0%)	

*Note:* Age is presented as mean (SD), and gender is presented as number (%). *p* < 0.05 is significant.

According to Table 2, there was no significant difference in the UNVA between the two groups at baseline (*p* = 0.864). Following the 30‐day administration of pilocarpine 1%, a significant reduction in the mean value of UNVA is reported in the intervention group (*p* ≤ 0.001). The most significant decrease is observed after an hour of treatment (*p* ≤ 0.001). There was a nonsignificant increase after 6 h (*p* = 0.297); however, the values decreased at 14 days and 30 days again. In contrast, no significant reduction is observed in the control group after 30 days (*p* = 0.063).

**TABLE 2:** The effect of pilocarpine 1% on uncorrected near visual acuity (UNVA) across different time points.

Time point	Control group	Intervention group	*p*	Treatment difference,% (95% CI)
Baseline	0.440 (0.137)	0.446 (0.133)	0.864	0.006 (−0.064, 0.076)
20 min	0.421 (0.147)	0.307 (0.134)	0.002	−0.114 (−0.187, −0.041)
An h	0.360 (0.159)	0.207 (0.126)	≤ 0.001	−0.153 (−0.225, −0.080)
6 h	0.423 (0.161)	0.246 (0.146)	≤ 0.001	−0.177 (−0.249, −0.104)
14 days	0.358 (0.166)	0.192 (0.121)	≤ 0.001	−0.166 (−0.239, −0.093)
30 days	0.343 (0.167)	0.172 (0.110)	≤ 0.001	−0.171 (−0.243, −0.098)
*p* (effect of time)	0.063	≤ 0.001		

*Note:* Values are presented as mean (SD) in Log MAR scale. *p* < 0.05 is significant.

**FIGURE 1:**
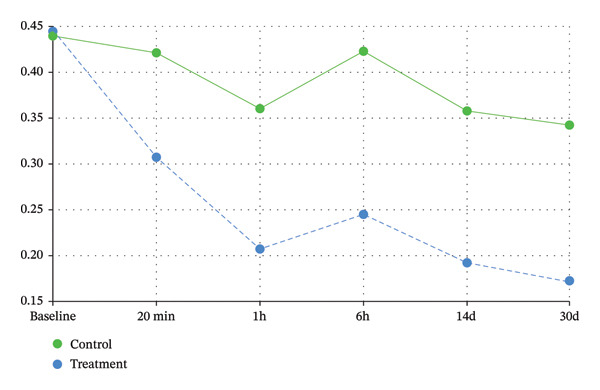
UNVA (Log MAR) scores for control and treatment groups over six time points.

The UNVA values were significantly lower in the intervention group compared to the control group at all time points after starting the treatment. The effect of intervention was statically significant (*p* ≤ 0.001), and the interaction of intervention and time was also significant (*p* = 0.032), indicating the variation of intervention’s effect on UNVA across the study period. Figure 1 presents the trend of mean UNVA scores across the six time points for both groups.

Table 3 presents the comparison of UDVA in the control and intervention groups over six time points. As shown, there is no significant difference between the two groups at the baseline (*p* = 0.503). Although the UDVA values were significantly higher in the intervention group compared to the control group at 20 min, an hour, 6 hours, and 14 days’ time points, there was no significant difference after 30 days of study between the two groups.

**TABLE 3:** The effect of pilocarpine 1% on uncorrected distance visual acuity (UDVA) across different time points.

Time point	Control group	Intervention group	*p*	Treatment difference,% (95% CI)
Baseline	0.720 (0.145)	0.700 (0.117)	0.503	−0.020 (−0.079, 0.039)
20 min	0.720 (0.145)	0.813 (0.097)	0.002	0.093 (0.035, 0.152)
An h	0.753 (0.117)	0.873 (0.069)	≤ 0.001	0.120 (0.061, 0.179)
6 h	0.720 (0.145)	0.827 (0.094)	≤ 0.001	0.107 (0.048, 0.165)
14 days	0.780 (0.124)	0.840 (0.081)	0.045	0.060 (0.001, 0.119)
30 days	0.787 (0.133)	0.840 (0.081)	0.074	0.053 (−0.005. 0.112)
*p* (effect of time)	0.161	≤ 0.001		

*Note:* Values are presented as mean (SD) in Log MAR scale. *p* < 0.05 is significant.

**FIGURE 2:**
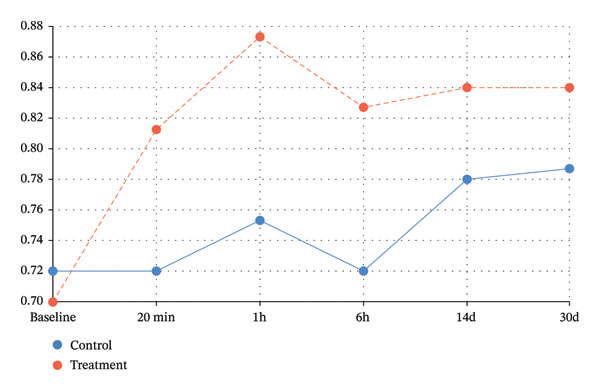
UDVA (Log MAR) scores for control and treatment groups over six time points.

Following the 30‐day administration of pilocarpine 1%, the UDVA showed a significant increase in the intervention group compared to the control group. The intervention group showed a significant time effect (*p* ≤ 0.001), while the control group did not (*p* = 0.161). The effect of the intervention was statistically significant (*p* ≤ 0.001), and the interaction of intervention and time was also significant (*p* ≤ 0.001), suggesting that the intervention’s effect on UDVA varied over time. However, the UDVA score was not significantly different between the two groups after a month (*p* = 0.074). Figure 2 demonstrates the trend of mean UDVA scores across the study for both groups.

Table 4 summarizes the adverse effects and satisfaction rates during the study. The adverse effects reported by the intervention group were statistically more than the control group, including eye pain, burning eye, and headache. However, no severe side effect was reported, and no patient discontinued the treatment. Also, patients’ satisfaction rates were significantly better in the intervention group (*p* ≤ 0.001).

**TABLE 4:** Adverse events and satisfaction rates following pilocarpine 1% administration after 30 days.

Variable		Control group	Intervention group	Total	*p*
Side effects	Non	30 (100.0%)	20 (66.7%)	50 (83.3%)	0.001
	Eye pain	0 (0.0%)	4 (13.3%)	4 (6.7%)	
	Burning eyes	0 (0.0%)	4 (13.3%)	4 (6.7%)	
	Headache	0 (0.0%)	2 (6.7%)	2 (3.3%)	

Satisfaction	Poor	14 (46.7%)	0 (0.0%)	14 (23.3%)	≤ 0.001
	Moderate	16 (53.3%)	16 (53.3%)	32 (53.3%)	
	Good	0 (0.0%)	12 (40.0%)	12 (20.0%)	
	Very Good	0 (0.0%)	2 (6.7%)	2 (3.3%)	

*Note:* Values are presented as number (%). *p* < 0.05 is significant.

## 4. Discussion

In this triple‐blinded randomized clinical trial, a total of 60 patients with presbyopia were divided into the two treatment and control groups, receiving pilocarpine 1% drops and placebo, respectively. In this prospective interventional study, the effect of 1% pilocarpine eye drops on both UNVA and UDVA of patients was investigated, while also evaluating any side effects or dissatisfaction of individuals over a month. The two groups initially had no statistically significant differences in terms of age, gender, UNVA, and UDVA.

After 1 month, the UNVA in the treatment group significantly improved from 0.45 ± 0.13 to 0.17 ± 0.11 log MAR, which is significantly better than the control group. Many other studies are in line with this result [2, 10, 11]. In the study conducted by Vargas et al., more than 90% of the patients experienced an improvement in near vision by two lines within 2 h [12].

Benozzi et al. indicated that this pharmacological treatment appears effective in achieving independence from spectacles for near visual tasks in individuals with presbyopia. It serves as an efficient solution for patients ranging from their 40 s to their 60 s [13]. However, these findings should be interpreted cautiously compared to the present study, as the formulations evaluated in those studies involved combination pharmacological regimens rather than pilocarpine 1% monotherapy.

In the present study, the greatest improvement in UNVA occurred within the first hour after instillation, which is consistent with the rapid onset of action reported in several other studies [10, 14]. There is concern that pilocarpine’s effects may be short‐lived due to potential neural adaptation or tolerance over time. However, this study’s findings demonstrated that after 30 days of daily use, the intervention group maintained significantly better UNVA than the control group. This sustained improvement aligns with the findings of Mousavi et al., who reported that pilocarpine can remain effective for up to a year [7].

In addition, it should be noted that the visual acuity assessments performed at the 30‐day follow‐up were obtained approximately one hour after drop instillation. Therefore, the observed visual outcomes reflect the effect of pilocarpine after 1 month of regular use under active treatment conditions rather than untreated baseline conditions.

Pilocarpine appears to enhance UNVA through multiple physiological mechanisms. It causes the pupils’ miosis, creating a pinhole effect that increases the depth of focus [15]. It also stimulates contraction of the ciliary muscle, improving the eye’s ability to accommodate [2, 16]. Vejarano et al. reported a mild myopic shift following pharmacological treatment for presbyopia, supporting the role of accommodative changes in improving near visual performance [17].

Interestingly, when pilocarpine is used at concentrations above 1%, it can shift the anterior lens and move the iris–lens diaphragm, further improving near vision [18]. Notably, these effects occur without significant changes in intraocular pressure, suggesting a selective action on accommodative structures [7].

In the current study, the treatment group experienced a significant decline in UDVA by Day 30 compared to their initial measurements. This finding is somewhat surprising, as many previous studies have reported stable distance vision during similar treatments [2, 19]. One possible explanation is the pharmacodynamic effect of sustained pupil constriction and ciliary muscle contraction, which may temporarily change the refractive state. In addition to improving near vision through increased depth of focus, ciliary muscle contraction may induce a state of pharmacologically induced pseudo accommodation [14].

While this mechanism may enhance near visual performance, it might also shift the focal point toward near distances, leading to a mild reduction in distance visual acuity in some individuals. However, no significant differences were observed between the treatment and the control groups at the final assessment. Therefore, the clinical significance of this finding remains uncertain and should be interpreted cautiously until confirmed by larger studies with longer follow‐up periods.

In line with previous studies, no significant side effects, such as retinal changes or increased IOP, were observed [2, 12, 14]. The stable IOP is aligned with the findings of Bennozi et al., who attributed it to the intrinsic pharmacodynamics of pilocarpine [20]. Some mild adverse effects were reported in the treatment group, including eye pain (13.3%), burning sensation (13.3%), and headache (6.7%). Nevertheless, none of these were severe enough to warrant discontinuation of treatment. Overall, the treatment was well tolerated, and there were no withdrawals from the study due to side effects.

Mousavi et al. reported that side effects resolved spontaneously within a few days [7]. Also, Bennozi et al. noted that headaches are the second most common side effect. Patients described the headaches as tolerable, with reports that they spontaneously resolved some minutes after the drops were administered, likely due to parasympathetic stimulation [13]. Nevertheless, Saxena et al. emphasized that healthcare professionals must constantly monitor and regularly assess patient responses to ensure safety and achieve optimal treatment outcomes [2].

One of the significant strengths of the present study is the structured approach to monitoring patients over time. Unlike many earlier studies with limited follow‐up, this research evaluated visual acuity at six different time points—baseline, 20 min, 1 h, 6 h, 14 days, and 1 month—providing valuable insights into the onset, peak effects, and duration of pilocarpine’s effects. Additionally, the triple‐blinded design, combined with systematic monitoring of side effects, further enhanced the reliability of the findings.

### 4.1. Limitation

This study has several limitations. First, the relatively high baseline UDVA values among participants may have influenced the observed changes in distance visual acuity during follow‐up. Unlike some previous studies that enrolled subjects with normal baseline distance vision, broader inclusion criteria were used in this study. Therefore, any changes in UDVA should be interpreted carefully.

Second, baseline ocular characteristics that may influence treatment response, including presbyopia severity and pupil size, were not assessed. Specifically, accommodative amplitude, near add power, pupillometry, and mesopic pupil size measurements were not obtained, limiting detailed subgroup analyses. In addition, the refractive status was not analyzed as a separate variable, and participants were not categorized according to baseline refractive error. Finally, spectacle independence and intermediate visual acuity were not specifically evaluated. Future research that includes these factors could provide a clearer picture of pilocarpine’s effectiveness in managing presbyopia.

## 5. Conclusion

Once‐daily administration of 1% pilocarpine eye drops significantly improved UNVA in presbyopic patients, with sustained efficacy over 30 days. While a reduction in UDVA was observed, it was still clinically acceptable. The treatment was well tolerated, with high patient satisfaction and only mild, infrequent adverse effects. These results suggest 1% pilocarpine as a safe and effective noninvasive option for managing presbyopia.

## Author Contributions

Conceptualization and writing, Fateme Heidari; formal analysis, Fateme Heidari; supervision and physical examiner, Mohsen Gohari; data collecting, Mohammad Hosein Abootorabi.

## Funding

The authors received no specific funding for this work.

## Disclosure

All authors have read and agreed to the published version of the manuscript.

## Consent

Informed consent was obtained from all patients involved in this study.

## Conflicts of Interest

The authors declare no conflicts of interest.

## Data Availability

Additional data used to support the findings of this study are available from the corresponding author upon reasonable request.
